# Exploring the interplay between angiosperm chlorophyll metabolism and environmental factors

**DOI:** 10.1007/s00425-024-04437-8

**Published:** 2024-06-11

**Authors:** Shunyuan Yong, Qian Chen, Fan Xu, Hao Fu, Guolu Liang, Qigao Guo

**Affiliations:** 1 Key Laboratory of Agricultural Biosafety and Green Production of Upper Yangtze River (Ministry of Education), College of Horticulture and Landscape Architecture, Southwest University, Chongqing, 400715 People’s Republic of China; 2 State Cultivation Base of Crop Stress Biology for Southern Mountainous Land of Southwest University, Academy of Agricultural Sciences of Southwest University, Chongqing, 400715 People’s Republic of China; 3 College of Agronomy and Biotechnology, Southwest University, Chongqing, 400715 People’s Republic of China

**Keywords:** Chlorophyll, Chlorophyll metabolism, Tetrapyrrole pathway, Angiosperm, Environmental effects

## Abstract

**Main conclusion:**

In this review, we summarize how chlorophyll metabolism in angiosperm is affected by the environmental factors: light, temperature, metal ions, water, oxygen, and altitude.

**Abstract:**

The significance of chlorophyll (Chl) in plant leaf morphogenesis and photosynthesis cannot be overstated. Over time, researchers have made significant advancements in comprehending the biosynthetic pathway of Chl in angiosperms, along with the pivotal enzymes and genes involved in this process, particularly those related to heme synthesis and light-responsive mechanisms. Various environmental factors influence the stability of Chl content in angiosperms by modulating Chl metabolic pathways. Understanding the interplay between plants Chl metabolism and environmental factors has been a prominent research topic. This review mainly focuses on angiosperms, provides an overview of the regulatory mechanisms governing Chl metabolism, and the impact of environmental factors such as light, temperature, metal ions (iron and magnesium), water, oxygen, and altitude on Chl metabolism. Understanding these effects is crucial for comprehending and preserving the homeostasis of Chl metabolism.

## Introduction

**Fig. 1:**
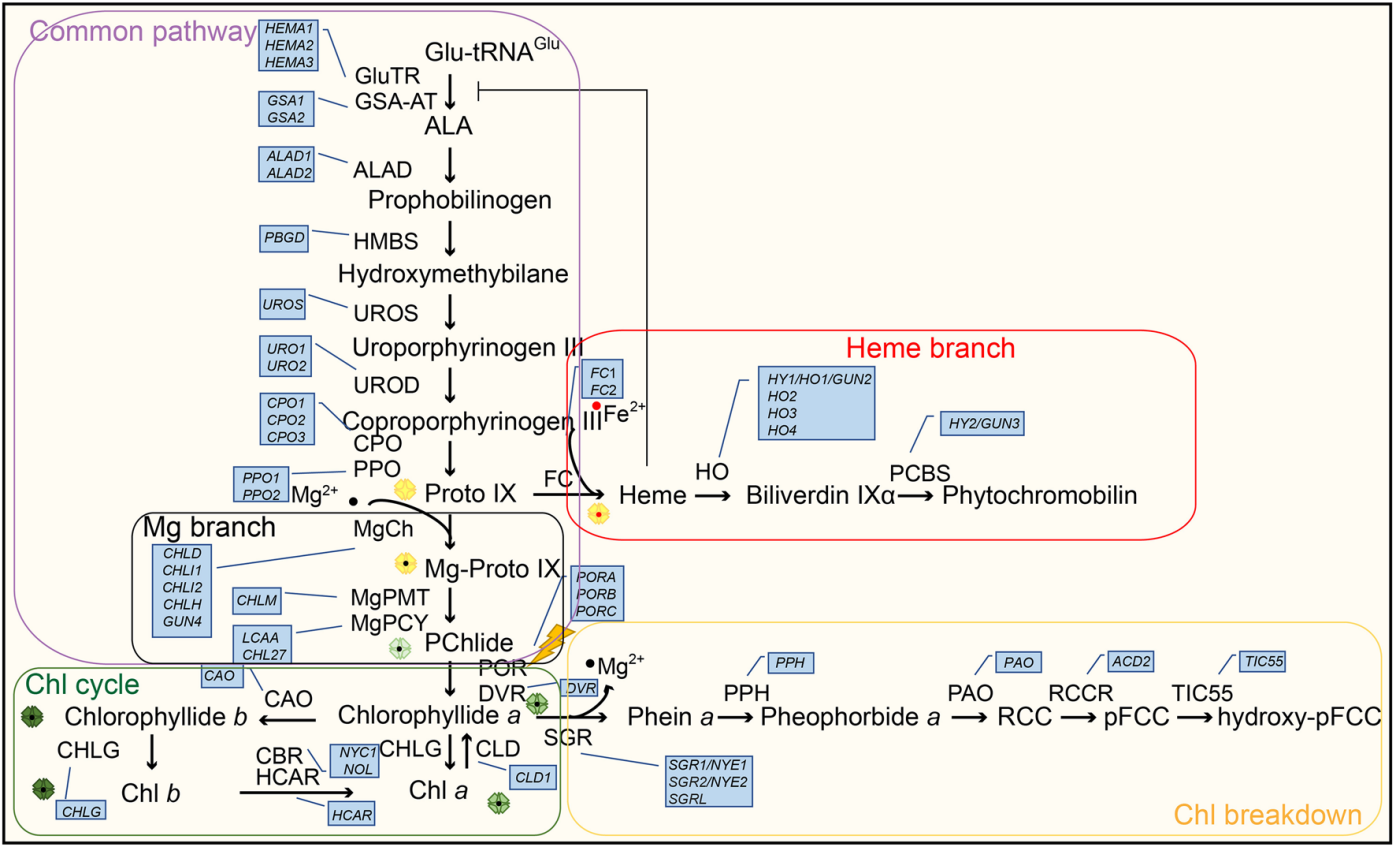
A schematic model of the chlorophyll (Chl) metabolic pathway, represented by *Arabidopsis thaliana*. Arrows indicate reactions. The enzymes involved are listed next to the arrows. The genes involved are in the blue boxes. Black dots represent Mg^2+^ and red dots represent Fe^2+^. Yellow prisms represent Proto IX. Light green prisms with black dot and yellow prism with red dot represent Mg-Proto IX and Heme, respectively. Green prisms with black dot represent Chlorophyllide *a* and Chl* a*, and dark green prisms with black dot represent Chlorophyllide *b* and Chl *b*

Chlorophyll (Chl) is the predominant pigment in the biosphere and plays a crucial role in the photosynthesis of angiosperms. The attainment of dynamic stability in Chl content relies on accurate and efficient Chl metabolism. The mechanisms underlying Chl synthesis and degradation during angiosperms development have been extensively investigated for several decades (Tanaka and Tanaka 2006; Wang et al. 2020; Wang and Grimm 2021). Previous studies on Chl metabolism have been extensively investigated, thus we will provide a concise overview here (Fig. 1). Chl synthesis is dependent on the tetrapyrrole biosynthesis (TPB) pathway (Brzezowski et al. 2015; Tanaka and Tanaka 2007; Vavilin and Vermaas 2002). In Arabidopsis (*Arabidopsis thaliana*), the initial step involves the rate-limiting enzyme glutamate 1-semialdehyde aminotransferase (GSA-AT) and glutamyl-tRNA reductase (GluTR), which convert glutamyl-tRNA^Glu^ into 5-aminolevulinic acid (ALA). A series of subsequent transformations converts ALA into protoporphyrin IX (Proto IX), a common precursor of Chl and heme. Proto IX becomes Magnesium-Proto IX (Mg-Proto IX) with Mg^2+^ in the presence of Mg-chelatase (MgCh), and Mg-Proto IX will lose Mg^2+^ to form protochlorophyllide (Pchlide). Subsequently, the light-dependent Pchlide oxidoreductase (LPOR, hereinafter referred to as POR) (Reinbothe et al. 2010; Nguyen et al. 2021) works with divinyl reductase (DVR) (Wang and Grimm 2021) to produce chlorophyllide (Chlide) *a*. Chl synthase (CHLG) then catalyzes the formation of the hydrophobic Chl *a* by integrating the phytyl pyrophosphate into Chlide *a* (Lin et al. 2014). Chl *a* can be further reversibly converted to Chl *b* by “Chl cycle”, and these newly synthesized Chl *a* and Chl *b* rapidly integrate into the Chl-binding proteins (CBPs) of the photosystem (PS)-light-harvesting complexes (LHC) (Wang and Grimm 2021). The first crucial enzyme in the Chl degradation pathway is MgCh [STAYGREEN/NON-YELLOWING (SGR/NYE)], as removal of the core Mg^2+^ from Chl *a* irreversibly leads to its destruction (Chen et al. 2016). During leaf senescence, Chl *a* dissociates from the pigment-protein complex and undergoes breakdown through the pheophorbide *a* oxygenase (PAO)/phyllobilin pathway. The conversion of Chl *b* to Chl *a* is essential for the Chl degradation, as only Chl *a* and phosphorous pheophorbide are accepted as substrates by both NYEs/SGRs and PAO (Kruse et al. 1995; Qian et al. 2016). The primary fluorescent Chl catabolite (pFCC) and hydroxy-pFCC, generated through a series of reactions, are transported from the chloroplast and subsequently enter the vacuole after modification. Plants do not accumulate red Chl catabolites (RCCs), which are rapidly reduced to pFCCs. Deficiency in PAO or RCCR can accelerate cell death due to the accumulation of substrates for these enzymes (Pružinská et al. 2003). The Chl metabolic pathway in plants is highly intricate, involving various enzymes and genes, playing an indispensable role in the normal growth and development of angiosperms.

Since angiosperms are unable to migrate, the environment has a significant influence on their Chl metabolism. Although Chl metabolism has some commonalities in other organisms, this review focuses on the intricate interplay between angiosperms physiology and environmental factors, including light, temperature, metal ions, water, oxygen, and altitude in order to maintain a delicate balance in Chl metabolism.

## Chlorophyll metabolism is dependent on Chl synthesis and degradation genes

Chl metabolism is dependent on the expression of numerous Chl biosynthesis genes (*CBGs*) and Chl catabolism genes (*CCGs*), and the enzymes and major genes involved in each important reaction step are enumerated in Fig. 1. The stay-green trait is observed in plants lacking Chl *b* reductase (CBR) (Sato et al. 2009), 7-hydroxymethyl Chl *a* reductase (HCAR) (Meguro et al. 2011), Mg dechelatase (SGR/NYE) (Chen et al. 2016) or pheophytinase (PPH) (Schelbert et al. 2009). In contrast to other Chl catabolic enzymes (CCEs), overexpression of SGR/NYE isoforms leads premature yellowing of leaves by promoting Chl breakdown in young leaves (Sakuraba et al. 2014b; Wu et al. 2016; Shimoda et al. 2016). The C-terminal cysteine-rich motif (Xie et al. 2019) of SGR/NYE1 is indispensable for its conserved role in Chl degradation (Qian et al. 2016). Studies have demonstrated that mutations in homologs of SGR1/NYE1 lead to the retention of a green phenotype in various plant species, including Arabidopsis (Ren et al. 2007), rice (Park et al. 2007), pea (Sato et al. 2007), tomato (Barry et al. 2008), and brassica napus (Qian et al. 2016). SGR contributes to the deterioration of the photosystem in addition to Chl degradation (Shimoda et al. 2016).

## Chlorophyll biosynthesis requires a balance of chlorophyll and heme

In the TPB pathway that occurs in plastids, a crucial heme branch exists where Mg (Mg^2+^) ions are inserted into Proto IX to generate Chl, while ferrous (Fe^2+^) ions are inserted into Proto IX to generate heme. The initiation of Chl production relies on MgCh, an ATP-dependent heteromeric polymerase composed of the catalytic H subunit (CHLH) and the two AAA+ proteins CHLD and CHLI (Axelsson et al. 2006). Upon the binding of the H subunit to the substrate Proto IX in complex with ATP-I-D-Mg^2+^, allosteric changes are induced in the D subunit, thereby activating the ATPase activity of the I subunit for ATP hydrolysis, which supplies energy for Mg chelation. Subsequently, this leads to the formation of a holoenzyme by chelating Mg^2+^ into Proto IX, resulting in the completion of the entire catalytic process. The CHLI mutation resulted in yellow-green strawberry leaves and reduced Chl levels. Under high light intensity (300 μmol m^−2^ s^−1^), the mutant exhibited impaired chloroplast development, decreased photosynthetic capacity, significantly reduced Mg-Proto IX, and impaired Chl biosynthesis. Interestingly, this mutant demonstrated a certain tolerance to low light (50 μmol m^−2^ s^−1^), with normal chloroplast development, increased Chl content, and higher photosynthetic capacity compared to the wild type (Ma et al. 2023). This suggests a potential effect of light on MgCh enzyme activity. Heterozygous barley mutants with deficiencies in MgCh also displayed decreased Chl biosynthesis (Braumann et al. 2014).

The conversion of Proto IX to heme is catalyzed by ferrochelatase (FC), and subsequently, heme is transformed into phytochromobilin (Tanaka and Tanaka 2007). Inhibition of FC1 expression results in a reduction in heme levels. However, this does not affect Chl levels or the performance of photosystem II (PSII) (Nagai et al. 2007). A high-Chl mutant *dg* was identified in a library of Chinese cabbage mutants, which maintains a fine-tuned balance between heme and Chl synthesis through a regulatory mechanism that enhances Chl synthesis via dBrFC2-mediated promotion of BrPORB enzymatic responses. An amino acid mutation in the Chl *a*/*b*-binding motif (CAB) of ferrochelatase 2 (BrFC2) in *dg* was observed to enhance the formation of BrFC2 homodimers, thereby promoting heme production. Both wild-type BrFC2 and mutant dBrFC2 interacted with Pchlide oxidoreductases B1 and B2 (BrPORB1 and BrPORB2), but dBrFC2 exhibited a higher affinity for the substrate Pchlide, consequently promoting an elevation in Chl content (Liu et al. 2022). The regulation of heme and Chl synthesis during TPB plays a crucial role in maintaining optimal Chl levels, ultimately enhancing photosynthetic efficiency.

## Post-translational coordination of chlorophyll synthesis and breakdown

The majority of research on Chl metabolism has primarily focused on transcriptional regulation. However, recent studies have made significant advancements in understanding post-translational processes. Several post-translational factors have been identified to regulate TPB, including GluTR-Binding protein (GBP) (Czarnecki et al. 2011), the chaperone Chloroplast Signal Recognition Particle 43 (cpSRP43) (Wang et al. 2018b), and LHC-LIKE 3 (Tanaka et al. 2010). GluTR is considered a pivotal regulatory enzyme that undergoes tight control at both transcriptional and post-translational levels. GBP (previously known as PROTON GRADIENT REGULATION7), a constituent of the thylakoid membrane's 300-kD protein complex, facilitates heme synthesis by retaining a fraction of GluTR on the plastid membrane responsible for ALA transportation into the heme biosynthesis pathway (Czarnecki et al. 2011). cpSRP43 mediates targeting and insertion of light-harvesting Chl *a*/*b*-binding proteins (LHCPs) and directly interacts with GluTR, indicating a crucial mechanism for post-translational coordination between LHCP insertion and Chl biosynthesis. Moreover, cpSRP43 and GBP exert distinct effects on the stability of GluTR (Wang et al. 2018b). It has been discovered that cpSRP43 interact with PORB, safeguarding its stability and normal functionality to ensure efficient Chl synthesis during leaf greening and heat shock responses (Ji et al. 2023). Although several post-translational control mechanisms regulating the Chl biosynthesis pathway have been characterized (Czarnecki and Grimm 2012; Brzezowski et al. 2015), further investigations are warranted to comprehensively elucidate the post-translational regulation of Chl catabolic metabolism, encompassing the activity, stability, and suborganelle localization of diverse genes associated with Chl metabolism (Hörtensteiner and Kräutler 2011; Kuai et al. 2018).

**Fig. 2:**
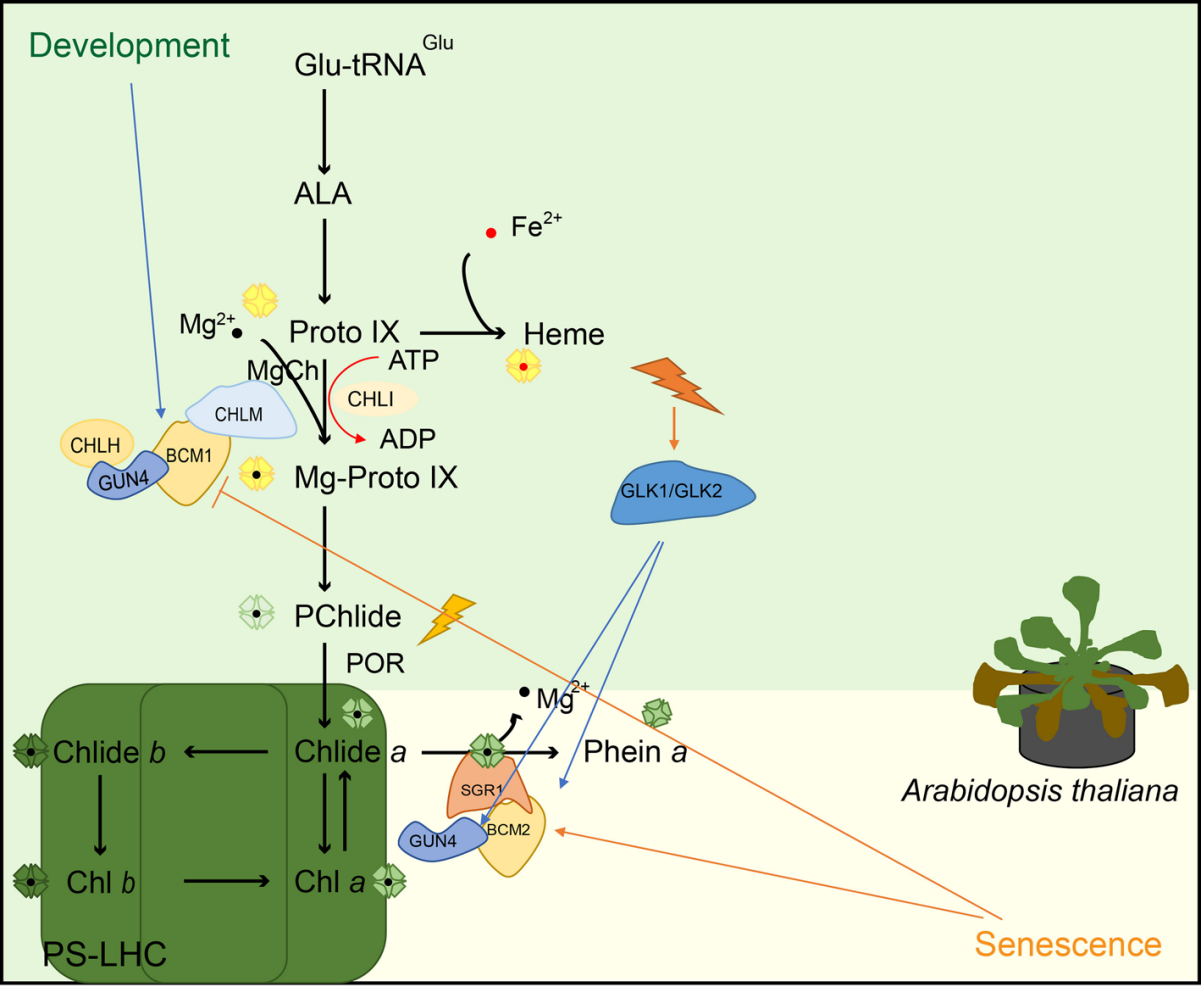
Post-translational coordination of Chl synthesis and breakdown in *Arabidopsis* leaves is regulated by GUN4. GUN4 interacts with CHLH, binds to the intermediates Proto IX and Mg-Proto IX, and activates Mg-chelatase (MgCh, consisting of the subunits CHLH, CHLI, and CHLD) as a post-translational regulator of Chl metabolism (Adhikari et al. 2011; Zhang et al. 2021). Early in the development of the leaf, BCM1 promotes MgCh through the action of GUN4, interacts with Mg-protoporphyrin IX methyltransferase (CHLM), thus promoting Chl biosynthesis, and interacts with SGR1, destabilizing SGR1 and preventing Chl decomposition (Wang et al. 2020; Yamatani et al. 2022). Newly synthesized Chl rapidly binds to various Chl-binding proteins in the photosystem (PS)-light-harvesting complex (LHC). BCM1 is severely suppressed during the start of leaf senescence, while BCM2 and SGR1 are upregulated. Although BCM2 prevents Chl catabolism, the large accumulation of SGR1 eventually leads to Chl catabolism in plants

The precise regulation of Chl levels is crucial for optimizing photosynthesis and plant adaptation, which relies on maintaining a balance between Chl anabolism and catabolism rates (Fig. 2). GENOMES UNCOUPLED 4 (GUN4) has emerged as a key post-translational regulator of Chl biosynthesis (Peter and Grimm 2009). GUN4 plays a role in plastidic retrograde signaling, binds to Proto IX and Mg-Proto IX, and activates MgCh (Larkin et al. 2003). Previously, it was believed that GUN4 enhances the interactions between CHLH and chloroplast membranes, which serve as the site of MgCh activity, by forming more stable connections with porphyrins (Adhikari et al. 2011). Overexpression of *GUN4* frequently results in the activation of enzymes, involved in Chl biosynthesis, whereas deficiency of *GUN4* hampers ALA synthesis and Chl accumulation (Peter and Grimm 2009). BALANCE OF Chl METABOLISM 1 and 2 (BCM1 and BCM2) regulate post-translational dynamics of Chl during leaf development (Wang et al. 2020). BCM1, an intrinsic membrane protein, plays conserved roles in both Chl metabolic pathways and is predominantly expressed during seedling growth. In response to light, BCM1 interacts with GUN4 to enhance MgCh activity and promote Chl biosynthesis (Zhang et al. 2020; Wang et al. 2020). Conversely, the expression of *BCM2* is upregulated during leaf senescence. BCMs are highly conserved regulators of Chl metabolism in plants, with their direct homologs being selected during the domestication of the tomato, soybean, and rice (Luquez and Guiamét 2001; Wang et al. 2018a). Studies have demonstrated that both BCMs function as negative regulators of Chl catabolism by interacting with SGR1/NYE1. Overexpression of either *BCM1* or *BCM2* delays Chl degradation and maintains a green phenotype, while inactivation of both BCMs result in a premature aging phenotype. Importantly, it should be noted that alterations in Arabidopsis' levels of *BCM* do not impact the expression of *CBGs* or *CCGs* (Wang et al. 2020).

## Imbalance of chlorophyll metabolism and the presence of antioxidant mechanisms

Reactive oxygen species (ROS) can be excessively generated in plants under abiotic or biotic stress, resulting in cellular damage (oxidative stress) and even cell death in severe cases (Apel and Hirt 2004). ROS are frequently produced because of various metabolic pathways in plants, including the Chl metabolic pathway, and ROS in turn destroy Chl molecules and/or Chl precursor (Chakraborty and Tripathy 1992; Santabarbara et al. 2002; Pattanayak and Tripathy 2011). The effects of light (e.g., high irradiance, fluctuating light, and short wavelengths of light) may damage photosynthetic proteins, leading to a decrease in photosynthetic efficiency and the formation of photodamage. The imbalance between PSII photodamage and its subsequent repair leads to photoinhibition. To prevent the accumulation of photodamaged PSII, photosynthetic organisms need to undergo repair, which involves the destruction and regeneration of D1 protein, and inhibit the repair of PSII by inhibiting the synthesis of PSII proteins (Takahashi and Murata 2008). Generally, rapid integration of Chl supply into CBPs occurs to prevent accumulation of phototoxic Chl and its metabolic intermediates (Allen et al. 2011). Given that both wavelength and photosynthetic photon flux density vary throughout the day, Chl biosynthesis predominantly takes place under low light conditions, while midday and early afternoon witness PSII photoinhibition and Chl degradation. Singlet oxygen (^1^O_2_) and superoxide (O_2_·^−^) are generated in chloroplasts by PSII and PSI, respectively, under conditions of excessive light stress (Asada 2006; Waszczak et al. 2018; Smirnoff and Arnaud 2019). Dogra and Kim (2020) focused on elucidating the mechanisms underlying ^1^O_2_ production and its impact on plant physiology, providing a comprehensive account of its generation and functional role within chloroplasts. Multiple potential sites for O_2_·^−^ generation in chloroplasts, including PSI, PSII, and the electron transport chain, have been identified; moreover, O_2_·^−^ can give rise to H_2_O_2_. A thorough review of H_2_O_2_ metabolism within chloroplasts is available in Smirnoff and Arnaud (2019). To avoid excessive accumulation of ROS in chloroplasts, Lutein and zeaxanthin, two carotenoids present in the LHC, regulate Chl's dynamic stability by quenching ^3^Chl* and preventing anomalous energy transfer to surrounding molecules. However, insufficient quenching of ^3^Chl* can lead to its reaction with molecular oxygen (^3^O_2_) released by the water-splitting event in the oxygen-producing complex (OEC), resulting in the formation of ^1^O_2_ (van Mieghem et al. 1995; Rinalducci et al. 2004). Under mild stress conditions, a fraction of ^1^O_2_ can evade the quenching mechanism in PSII and diffuse into the thylakoid membrane, resulting in lipid peroxidation. To counteract this process, tocopherol and plastoquinone (PQ), both present in the thylakoid membrane, act as preventive measures by detoxifying ^1^O_2_ (Santabarbara et al. 2007; Li et al. 2009). Additionally, in the stroma, ascorbate can eliminate released ^1^O_2_ from the thylakoid membrane (Kruk and Trebst 2008). Plastochromanol-8 (PC-8), an efficient scavenger of ^1^O_2_, efficiently safeguards lipids against photooxidative damage in Arabidopsis plants (Szymańska and Kruk 2010; Rastogi et al. 2014). Metabolomics research has further demonstrated that three flavonoid metabolites exclusively found in late-maturing soybean seeds (farrerol-7-*O*-glucoside, cyanidin-3-*o*-(6′-*o*-feruloyl) glucoside, and kaempferide-3-*o*-(6′-malonyl) glucoside) can effectively mitigate Chl degradation by scavenging oxygen free radicals within the chloroplasts (Wang et al. 2022a). However, there is still a need to explore additional mechanisms involved in maintaining equilibrium between Chl and ROS.

## Light: a key environmental factor for chlorophyll balance

Light plays a significant role in plant growth and development as both a developmental switch and an information source, modulating multiple crucial events involved in plant development (de Wit et al. 2016). Light also exerts an impact on Chl metabolism in plants, resulting primarily in variations of leaf color. A study conducted on 14 gold leaf ornamental plants revealed that the variations in ambient light intensity significantly influenced the color of gold leaf plant leaves, resulting in an observed increase in Chl content and the Chl/carotenoid ratio as the leaf position decreased (Yuan et al. 2010). Light-induced de-etiolation triggers chloroplast differentiation, and the presence of intact chloroplast structure is a prerequisite for Chl metabolism. A comprehensive review of etiolation and de-etiolation studies has been previously conducted (Armarego-Marriott et al. 2020). While chloroplasts efficiently capture light energy and autonomously adapt to changes in light conditions through diverse biochemical mechanisms (Kovács et al. 2006; Johnson et al. 2011; Nath et al. 2013; Wolf et al. 2020), photoreceptors including phytochromes (Phys), cryptochromes (Crys), ultraviolet-B resistance 8 (UVR8), along with other regulatory proteins regulating plant transcriptomic responses, perceive alterations in both light intensity and quality (Liscum et al. 2020; Cheng et al. 2021). Arabidopsis possesses at least five classes of wavelength-specific photoreceptors that perceive distinct light signals: Phys (phyA-phyE) detect red and far-red light, Crys (CRY1 and CRY2), phototropins (PHOT1 and PHOT2), as well as members of the ZEITLUPE family (ZTL, FKF1, and LKP2) sense ultraviolet (UV)-A and blue light; while UV-B RESISTANCE LOCUS 8 (UVR8) absorbs UV-B radiation (Paik and Huq 2019; Podolec et al. 2021; Cheng et al. 2021). The coordination of multiple photoreceptors and their internal signaling pathways in plants has been summarized, highlighting their regulation of various downstream responses at the molecular and physiological levels to enable adaptation and survival in a changing environment (Paik and Huq 2019).

Similar to other eukaryotes, plant gene expression is regulated at multiple levels, including chromosomal organization, transcriptional control, post-transcriptional modifications, and translational processes (Cazzonelli et al. 2020). At the transcriptional level, light-induced regulation of plant genes plays a pivotal role through various cis-acting elements such as G-box (Ezer et al. 2017; Suekawa et al. 2018), E-box (Liu et al. 2015), D-box (Guo et al. 2016) and F-box (Lee et al. 2017). Numerous crucial transcription factors involved in light-related events, such as *ELONGATED HYPOCOTYL5* (*HY5*) (Bae and Choi 2008), *PHYTOCHROME-INTERACTING FACTORs* (*PIFs*) (Leivar and Quail 2011), and *GOLDEN2-LIKE* (*GLK*) (Powell et al. 2012; Wang et al. 2013), regulate chloroplast biogenesis and photomorphogenesis. HY5, upon exposure to UV-B radiation and visible light, binds to its gene promoter facilitating gene expression (Abbas et al. 2014; Binkert et al. 2014), while being downstream regulated by GUN5 and Heat-shock protein HSP90 for Chl biosynthesis regulation (Kindgren et al. 2012). PIFs directly bind to the promoter of the *GLK1* gene under dark conditions leading to the inhibition of *CBG* expression. It has been demonstrated that PIF4/5 can activate *CCG* expression during dark-induced leaf senescence either directly or indirectly (Sakuraba et al. 2014a; Song et al. 2014). Conversely, PIF proteins undergo rapid degradation in response to light, while activated GLK1 and GLK2 transcription factors positively regulate the expression of photosynthesis-related nuclear genes and *HEMA1*, *CUN4*, *CHLH*, and *CAO*, thereby promoting Chl biosynthesis (Waters et al. 2009; Martín et al. 2016). In angiosperms, the metabolic flow of Chl biosynthesis is regulated at the step of ALA synthesis. A negative feedback loop mediated by heme and Pchlide at the GluTR level reduces 5-ALA synthesis in darkness. In Arabidopsis, SUPPRESSOR OF OVEREXPRESSION OF CO 1 (SOC1), a trans-regulator of PPH, inhibits dark-induced oxidative stress and leaf senescence (Chen et al. 2017b). These findings collectively demonstrate that both light and darkness exert direct or indirect regulatory effects on Chl metabolism.

The TPB pathway involves a crucial light-dependent enzyme known as POR, which catalyzes the conversion of Pchlide to Chlide upon exposure to light (Gabruk and Mysliwa-Kurdziel 2015). The atomic structure of POR assemblies and its coordination with photosynthetic membrane biogenesis and Chl synthesis in plants have been reported (Nguyen et al. 2021). In the absence of light, POR filaments directly induce high-curvature tubules in the membrane with spectral properties resembling prelaminar bodies, whose light-induced decomposition provides lipids essential for thylakoid assembly (Nguyen et al. 2021). In Arabidopsis, three genes, namely *PORA*, *PORB*, and *PORC*, encode for the enzyme POR. *PORA* is expressed during the initial stage of greening, while *PORB* primarily maintains Chl levels throughout angiosperm development (Masuda et al. 2003). *PORC* expression levels were found to decrease in the dark and increase in the light (Wang et al. 2022b). The expression of POR displays distinct patterns in barley (Apel 1981) and cucumber (Fusada et al. 2000), implying the potential for diverse light regulation of these genes.

**Fig. 3:**
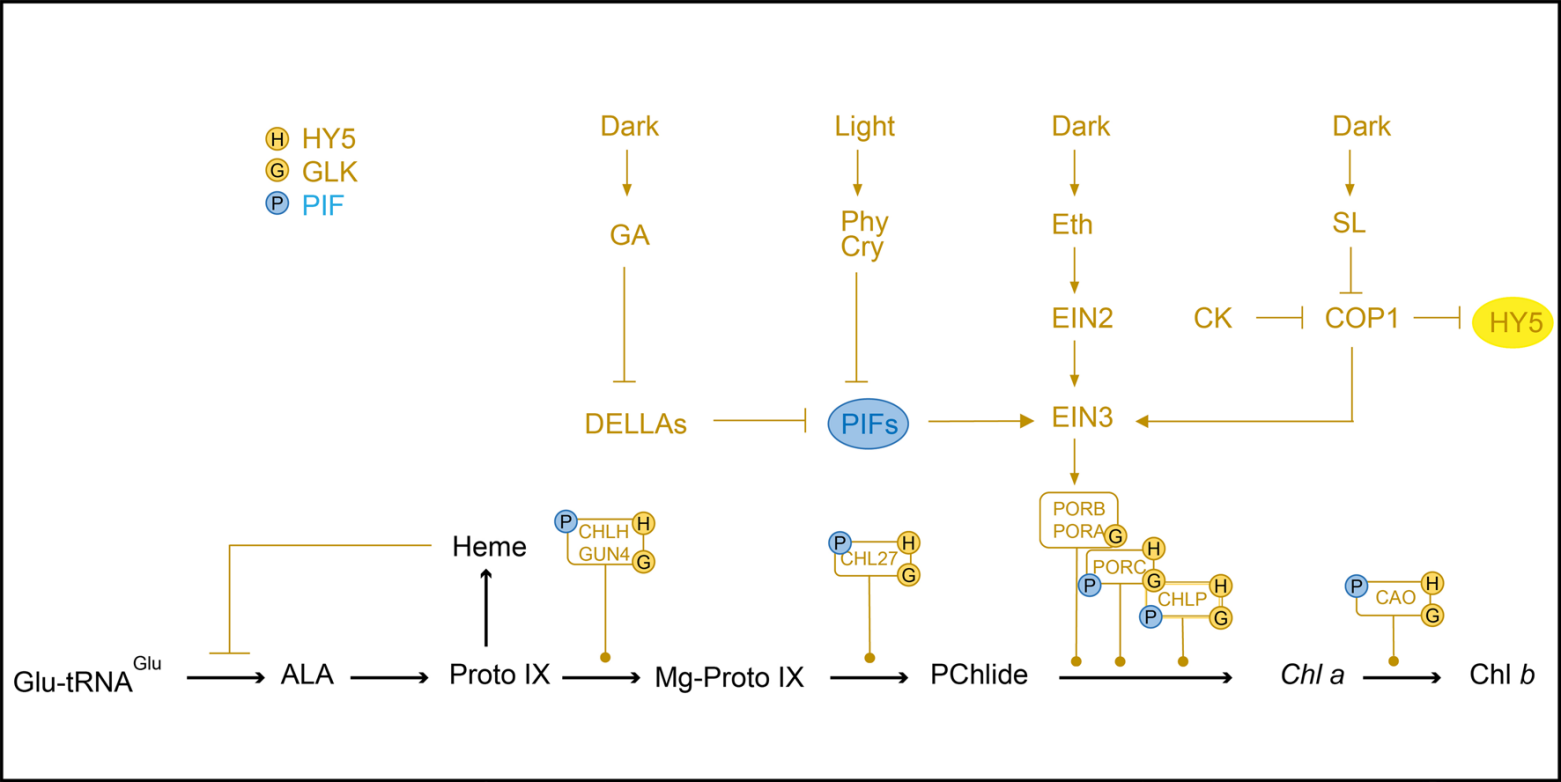
Effect of the hormone network, influenced by light, on Chl metabolism. Many important tetrapyrrole biosynthesis (TPB) genes, including CHLH, GUN4, and CAO, are upregulated as a result of the photosensitive and cryptochrome (Cry) photoreceptor families (de Wit et al. 2016; Kobayashi and Masuda 2016). Under light, Phy induces phosphorylation, ubiquitination, and subsequent degradation of PHYTOCHROME-INTERACTING FACTORs (PIFs), leading to the disinhibition of the TPB genes. By destroying DELLA proteins (DELLAs) and freeing PIFs from DELLA inhibition, accumulating gibberellin (GA) in the dark aids in the activation of PIFs (Cheminant et al. 2011; de Wit et al. 2016). The EIN proteins are stabilized by ethylene (Eth) and COP1 signals, and then the PORA and PORB are upregulated by binding to their promoter region (Zhong et al. 2009). HY5 protein is degraded in the dark by COP1-mediated ubiquitination(Xu 2020). At the same time, light mediated the accumulation of HY5 protein by blocking COP 1 activity and induced the expression of GLKs, which together upregulated key TPB genes. In addition, cytokinin (CK), strigolactone (SL). and lactones inhibited COP 1 activity and increased HY5 protein levels(Vandenbussche et al. 2007; Kobayashi et al. 2014; Zhang et al. 2024). Positive and negative regulation is depicted by arrows and bars, respectively

A point of interest is that light-built hormone networks have important effects on Chl metabolism. Modifications in hormone metabolism and transport mediate various light responses in plants (Kami et al. 2010). The intricate relationship between light perception and hormonal regulation in key life events has been extensively discussed (de Wit et al. 2016). Specifically, there is a growing understanding of the impact of light-induced hormone networks on Chl metabolism (Fig. 3). Research has delved into the PIF-auxin pathway in Arabidopsis seedlings, which is triggered by the deactivation of phytochrome B (phyB) due to a low red to far-red light ratio (R:FR) (Fernández-Milmanda and Ballaré 2021). PIFs play a pivotal role in repressing Chl production under dark conditions, acting as a central signaling hub that integrates light, hormonal signals, and various developmental cues (Leivar and Quail 2011). PIFs are indispensable for the negative regulation of genes involved in TPB pathway responsible for Chl synthesis. The accumulation of dark-accumulating gibberellins (GAs) contributes to PIF activation by degrading DELLA proteins (DELLAs) and relieving their inhibitory effects (Cheminant et al. 2011). Despite their ability to inhibit PIF activity through direct protein-protein interactions, DELLAs independently regulate the expression of *PORA* and *PORB* (Feng et al. 2008; de Lucas et al. 2008). In the absence of light, nuclear accumulation of PIFs suppresses photomorphogenic reactions, including Chl biosynthesis. Key regulators in ethylene (Eth) and abscisic acid (ABA) signaling pathways such as EIN3, ABI5, and ENHANCED EM LEVEL (EEL), are directly activated by PIF4 and PIF5 proteins. These aforementioned PIF proteins have previously been implicated in dark-induced leaf senescence (Sakuraba et al. 2014a; Song et al. 2014; Seaton et al. 2015; Liebsch and Keech 2016). EIN3/EIL1 and PIF1 directly bind to the promoters of POR genes, thereby activating their expression and regulating several TPB genes to prevent the accumulation of Pchlide, a phototoxic intermediate in Chl biosynthesis. By eliciting EIN3/EIL1 activation through its established signal transduction cascade, Eth enhances seedling greening (Zhong et al. 2009). In Arabidopsis cytokinin (CK) receptor mutants, the light-induced upregulation of *HEMA1*, *CHLH*, and *CHL27* was attenuated. However, treatment with CK in dark-grown wild-type seedlings promoted the expression of these genes (Hedtke et al. 2012; Kobayashi et al. 2014). CK and strigolactone (SL) have been shown to elevate HY5 protein levels while inhibiting COP1 activity (Vandenbussche et al. 2007; Tsuchiya et al. 2010). The pivotal role of plant hormones in governing growth and developmental processes is indisputable; however, further investigations are warranted to elucidate the intricate interplay between environmental conditions and hormonal regulation.

## Temperature: effects of high and low temperatures on chlorophyll metabolism

The significance of environmental temperature for plants is self-evident. On one hand, the impact of repeated and more frequent high temperatures in tropical regions on plant growth and development has raised growing concerns (Li et al. 2023; Zhu et al. 2023). High temperature accelerates Chl breakdown by upregulating genes associated with Chl degradation. Studies conducted on cucumbers have demonstrated that elevated temperatures induce the expression of *ABI5* and *MYB44*, thereby leading to Chl degradation. ABI5 directly binds to the promoters of *PPH* and *PAO*, thereby enhancing their expression and accelerating Chl degradation. The interaction between ABI5 and MYB44 relieves the transcriptional repression of *PPH* and *PAO*, leading to a reduction in MYB44’s binding to the promoters of *PPH* and *PAO*, ultimately resulting in MYB44 degradation (Liu et al. 2023a). On the contrary, low temperatures impose a significant environmental constraint that restricts plant development and crop productivity (Khan et al. 2017; Ding and Yang 2022). Low-temperature stress adversely affects photosynthetic capacity and efficiency by influencing gas exchange, pigment content, and chloroplast growth, resulting in reduced Chl fluorescence (Farooq et al. 2009). In postharvest tea leaves, cold-induced withering inhibits Chl breakdown through a substantial reduction in the activity and gene expression of Mg-dechelatase, Chlase, and pheophorbide an oxygenase enzymes (Yu et al. 2019).

Similar to light conditions, temperature conditions are intricately associated with the formation and development of chloroplasts, which serve as the site for Chl synthesis (Schwenkert et al. 2022). Both high and low-temperature stresses have detrimental effects on normal chloroplast formation, thereby impacting Chl metabolism. A genetic module known as TT3.1–TT3.2 has been identified as a regulator of heat sensitivity, ensuring protein quality in chloroplasts and promoting heat tolerance (Zhang et al. 2022). In recent years, more attention has been given to the effect of temperature on Chl breakdown rather than its regulation of Chl synthesis, which is often linked to light signals. The temperature response, including thermomorphogenesis, is also governed by phytochrome; specifically PhyB acts as a thermal sensor that regulates the temperature response (Casal and Balasubramanian 2019). The COP1-HY5 regulatory center has been demonstrated to play a crucial role in enhancing plant freezing tolerance, as well as accurately regulating plant responses to diurnal changes. Cold-induced translocation of COP1 from the nucleus to the cytoplasm results in an accumulation of HY5, which activates CBF1 and subsequently triggers the activation of multiple cold-responsive genes (Catalá et al. 2011). It is worth noting that light and temperature, two external environmental factors, often act synergistically as signals for regulating plant growth and development. Furthermore, their impacts on Chl metabolism frequently intersect and mutually influence each other.

## Metal ions: stress on chlorophyll metabolism by deficiencies of Fe and Mg

Approximately, 80% of leaf iron content is localized within chloroplasts, and plants cultivated in iron-deficient environments exhibit symptoms of chlorosis (Nam et al. 2021), with genes associated with photosynthesis and tetrapyrrole metabolism being extensively downregulated (Rodríguez-Celma et al. 2013). The development of Fe deficiency-induced chlorosis is contingent upon the effectiveness of phosphorus. Iron limitation leads to a downregulation of photosynthetic genes in a P-dependent manner, including PHT4, which encodes the chloroplast ascorbate transporter protein, and bZIP58, which encodes a nuclear transcription factor. These genes prevent the downregulation of photosynthesis genes and result in a stay-green phenotype under Fe–phosphorus deficiency (Nam et al. 2021). Optimal concentrations of iron fertilizers have the potential to restore functionality to the photosynthetic electron transport chain, enhance electron transfer efficiency, and facilitate balanced energy allocation (Gao et al. 2022). It is noteworthy that iron–sulfur [Fe–S] clusters in the photosynthetic electron chain can act as cofactors for some of the enzymes of Chl metabolism (Hauenstein et al. 2016; Lu 2018; Schmidt et al. 2020). Doubtlessly, Fe is an important metal in plant growth related to chloroplast function and efficient photosynthesis, and its importance along with manganese and copper has been reviewed in detail by Schmidt et al.(2020). Furthermore, the distribution of potassium and calcium ions in ilr3-4 leaves was also affected (Akmakjian et al. 2021). Recently, it has been reported that the calcium-dependent protein kinase CPK21/23 promotes iron uptake in plants by activating the iron transporter protein IRT1 during low iron stress conditions (Wang et al. 2023). Red light triggers the activation of calcium-dependent protein kinase CPKs via PhyB, leading to an increase in cytoplasmic calcium concentration. The red light-induced calcium signal is precisely transduced into the activation of intracellular light response genes by CPKs, which interact with red light-activated PhyB and phosphorylate it in the nucleus (Zhao et al. 2023). However, whether calcium signaling affects Chl metabolism through iron remains unknown.

As the central atom of Chl, Mg plays an important role, and replacing Mg with metals such as copper or cadmium has a toxic effect on Chl (Sarkar et al. 1994; Grajek et al. 2020). Mg^2+^ signals the adenylate (ATP+ADP+AMP) state of the cell, and most enzymes requiring ATP use the chelated form of ATP (MgATP) (Voon et al. 2018). Mg in chloroplasts is 20% of total Mg, but may increase to 50% under Mg-deficient or low-light conditions (Kleczkowski and Igamberdiev 2021). Insufficient Mg levels result in Chl degradation, characterized by intervein greening. Transcript levels of the gene encoding Chl* a*/*b*-binding protein 2 (CAB2) were downregulated within just 3 days after Mg deficiency was induced (Ogura et al. 2020). Comparative analysis of transcriptional and translational differences under early Mg deficiency revealed that mutants of the transcription factor HY5, H+/CATION EXCHANGER 1 and 3 (CAX1 and CAX3), and UBIQUITIN 11 (UBQ11) exhibited an earlier onset of yellowing phenotype under Mg deficiency (Li et al. 2021).The *cbd1* mutant exhibited reduced Chl content and lower Mg levels in the thylakoid compared to the wild type. Additionally, both *cbd1* and *cbd1gun5* mutants showed an accumulation of Mg-Proto IX. CBD1/BCM1 was shown to regulate chloroplast Mg homeostasis. CBD1/BCM1 was shown to regulate chloroplast Mg homeostasis. CBD1 was verified to have Mg transport activity using a Salmonella mutant strain MM281, which lacks the Mg2+ transport system (Zhang et al. 2020). However, further exploration is needed to understand how other Chl metabolism-related genes respond to Mg stress, particularly transcription factors and genes encoding Mg transporter proteins.

## Water content: drought stress causes damage to chlorophyll metabolism

Droughts, floods and other disasters can exert stress on plant growth. To address these challenges, a 5G breeding approach (Genome assembly, Germplasm characterization, Gene function identification, Genomic breeding, and Gene editing) has been proposed (Varshney et al. 2020). Transcriptome analysis of two broomcorn millet varieties with differing drought tolerance revealed downregulation of MgCh subunit D (ChlD) expression under drought stress which resulted in decreased efficiency of Mg-protoporphyrin IX production. In addition, enzymes related to Chl *a* and Chl *b*, such as CHLG and CAO, were downregulated. Conversely, the majority of genes involved in Chl degradation, including SGR, PAO, RCCR, and NOL, exhibited significant upregulation (Yuan et al. 2022). WRKY transcription factors play crucial roles in drought tolerance by regulating ROS scavenging enzymes, promoting xylem vessel development and stimulating cellulose and lignin synthesis in roots (Cai et al. 2014; Chen et al. 2017a; Zhao et al. 2018; Geng et al. 2018). In apple, MdWRKY17 has been demonstrated to effectively maintain optimal Chl levels and photosynthetic activity during drought stress by indirectly downregulating MdCLH, MdPAO, and MdRCCR while directly activating the transcription of MdSUFB, a crucial protein involved in Fe–S cluster assembly, Chl metabolism, and photosynthesis (Couturier et al. 2013; Shan et al. 2021). The phosphorylation of MdWRKY17 by the water-deficit-activated cascade of MdMEK2-MdMPK6 has been identified as indispensable for regulating the expression of MdSUFB to sustain Chl levels under mild water-deficit stress, as supported by knockdown experiments (Shan et al. 2021). Applying alpha lipoic acid, a cofactor for pyruvate dehydrogenase and glycine decarboxylase, which are specific mitochondrial enzyme complexes, to maize seedlings experiencing drought stress enhanced their Chl content and PSII activity. In alpha lipoic acid-treated seedlings under stress, the relative expression levels of the RCA and Mg-CHLI genes were significantly elevated, while the relative expression of the Chlase genes was significantly reduced compared to untreated seedlings (Sezgin et al. 2019). These findings suggest that drought stress can expedite leaf senescence and facilitate Chl degradation in various plant species. However, further investigations are warranted to explore strategies aimed at mitigating this phenomenon.

## Oxygen and altitude: an oxygen-sensing mechanism for angiosperm adaptation to altitude

Adaptation of individual plants (and populations) to survive at high altitudes is crucial, and it is argued that altitude adaptation involves direct perception of oxygen concentration across different altitudinal ranges. Studies have demonstrated that the regulation of hypoxia-related genes and the steady-state level of Pchlide, a biochemical intermediate in Chl biosynthesis, are controlled by an oxygen-sensing system in response to the local environmental absolute O_2_ concentration among various species along different altitudes (Abbas et al. 2022). In wild populations of Arabidopsis, *Solanum habrochaites*, *Solanum cheesmaniae*, and *Brachypodium distachyon* growing at various altitudes from sea level to over 3,000 m, a positive correlation between Pchlide accumulation and altitude is observed. Stable Pchlide levels play a crucial role in regulating Chl synthesis, and FLU has been demonstrated to impact Chl supply under light conditions (Hou et al. 2019). The modulation of Pchlide synthesis by O_2_ occurs through ErfVII-mediated GluTR inactivating complex components, specifically targeting the negative regulator FLU, via the PLANT CYSTEINE OXIDASE (PCO) N-degron pathway. This adaptive mechanism enables plants to respond to local atmospheric O_2_ levels and potentially prevent compromising singlet oxygen ROS production associated with light exposure. Furthermore, it has been observed that materials cultivated at higher altitudes and in an oxygen concentration of 15% exhibit reduced transcript levels of PORA, PORB, and CHLM genes compared to those grown under 21% oxygen conditions (excluding CHL27 or HEMA1) (Abbas et al. 2022). These findings emphasize the significant effects of altitude and oxygen levels on the expression of genes related to Chl metabolism, but the effects of oxygen partial pressure and UV radiation levels, which change with altitude, require further discussion. As mentioned earlier, imbalances in Chl metabolism are often associated with ROS, and perhaps the decrease in oxygen partial pressure reduces the photo-oxidative bleaching of Chl pigments. However, there is still much to uncover regarding transcriptional regulation in response to environmental cues.

## Conclusion

**Fig. 4:**
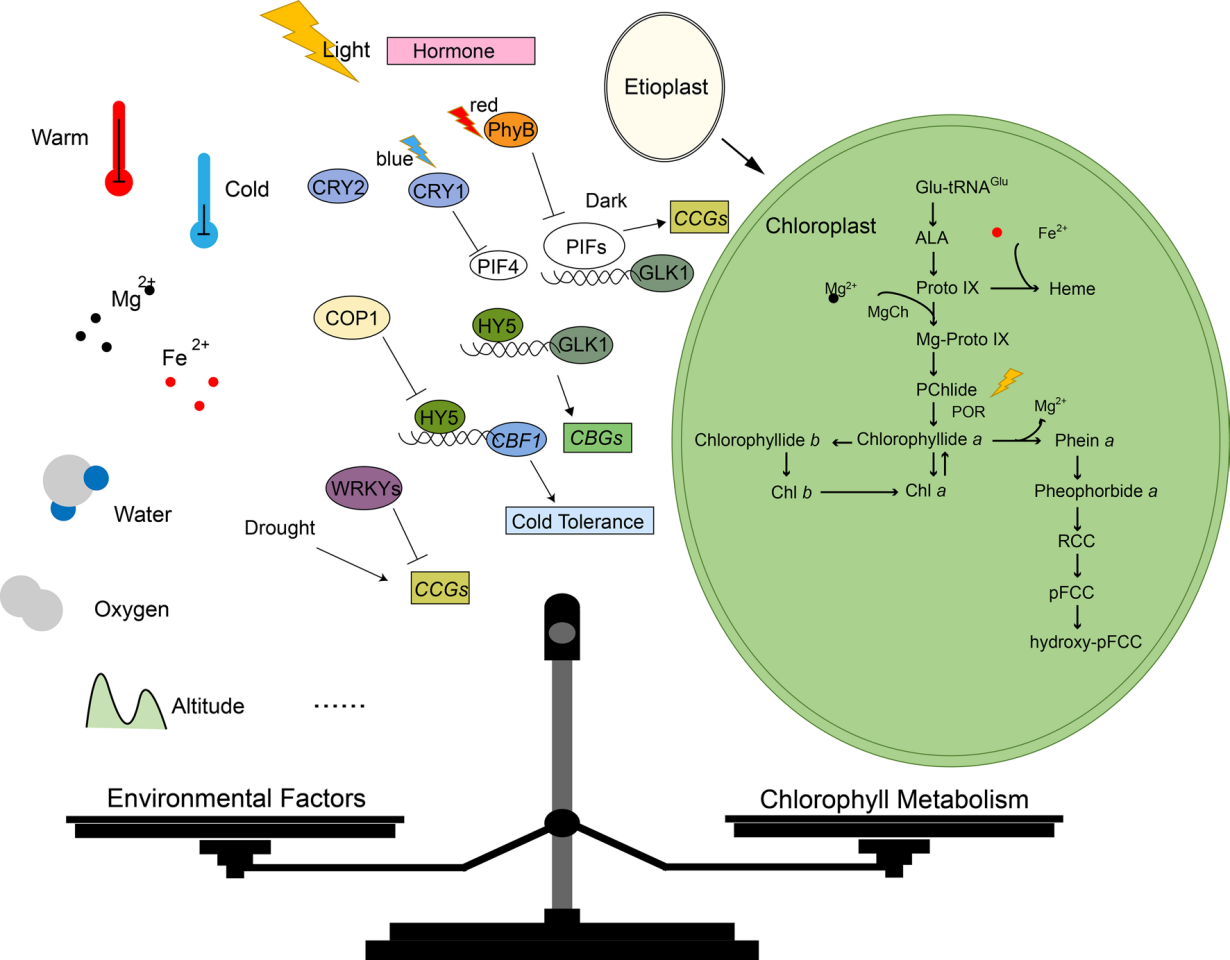
A schematic model outlining the homeostasis of Chl metabolism with environmental factors. PhyB senses red light and represses PIFs. PIFs in the dark inhibits GLK1, which directly or indirectly regulates *CCGs*. PhyB degrades PIFs in the light, and the activation of downstream target genes by HY5 is able to regulate Chl synthesis. PhyB plays a key role in the regulation of low-temperature signaling, while PIF4 is important for signaling in warmer environments. Mg^2+^ and Fe^2+^ participate in Chl synthesis as important elements in the heme branch. Water, oxygen, and altitude, can affect the expression of *CBGs* and *CCGs*

For angiosperms, Chl metabolism is not free from environmental influences, and here we summarize the topics of this review (Fig. 4). Light plays a crucial role in angiosperm morphogenesis and is the environmental factor that has received the most extensive attention in the field of Chl metabolism research. Although much progress has been made at the transcriptional level, more inputs are needed on how light affects Chl metabolism processes in networks with other important conditions (such as hormones, as described earlier) (Kami et al. 2010; Gabruk and Mysliwa-Kurdziel 2015; de Wit et al. 2016; Liu et al. 2017). In addition, the effect of alternating light and dark on protein localization and conformational changes still has great research potential. It is also interesting to note that temperature, together with light, is a network of signaling mechanisms that regulate plant growth and development, typically PhyB can be used as a thermal sensor to control temperature response (Casal and Balasubramanian 2019). Both Fe and Mg play indispensable roles in plant Chl metabolism (Li et al. 2021; Nam et al. 2021; Liu et al. 2023b). We believe that BCM1, which has only recently been revealed, appears to be closely related to Mg^2+^ (Zhang et al. 2020), but the upstream transcription factors on which it relies are not yet conclusive. Drought stress can expedite leaf senescence and enhance Chl degradation in a diverse range of plant species (Maroco et al. 2002; Shan et al. 2021; Yuan et al. 2022), and considering epigenetic influences on this process may contribute to a breakthrough in understanding. Furthermore, altitude and oxygen levels significantly impact the expression of genes associated with Chl metabolism (Abbas et al. 2022), but this environmental condition has been less well studied and the complexity of the environmental impacts can never be ignored. In the future, a better understanding of the complex interactions between Chl metabolism and environmental factors in angiosperms (and even in the biosphere as a whole) will be crucial for researchers to further explore and modify plants to improve yield and stress resistance.

## Data Availability

Not applicable.

## Abbreviations

Glu-tRNA^Glu^: l-Glutamyl-tRNA^Glu^
ALA: 5-Aminolaevulinic acid
ALAD: 5-Aminolaevulinic acid dehydratase
CAO: Chlide *a* oxygenase
CBGs: Chl biosynthesis genes
CBPs: Chl-binding proteins
CBR: Chl *b* reductase
CCEs: Chl catabolic enzymes
CCGs: Chl catabolism genes
CHLG: Chl synthase
Chlide: Chlorophyllide
CLD: Chl dephytylase
CPO: Coproporphyrinogen III oxidase
Crys: Cryptochromes
DVR: Divinyl reductase
GBP: GluTR-binding protein
GluTR: Glutamyl-tRNA reductase
GSA-AT: Glutamate 1-semialdehyde aminotransferase
HCAR: 7-Hydroxymethyl Chl a reductase
HMBS: Hydroxymethybilane synthase
LHC: Light-harvesting complexs
LHCPs: Light-harvesting Chl *a*/*b*-binding proteins
LPOR: Light-dependent protochlorophyllide oxidoreductase
MgCh: Mg-chelatase
MgPCY: Mg-protoporphyrin IX monomethylester cyclase
MgPMT: Mg-protoporphyrin IX methyltransferase
Mg-Proto IX: Magnesium-Proto IX
PAO: Pheophorbide *a* oxygenase
PCBS: Phytochromobilin synthase
Pchlide: Protochlorophyllide
pFCC: Primary fluorescent Chl catabolite
Phein * a *: Pheophytin *a*
Phys: Phytochromes
PPH: Pheophytinase
PPO: Protopoiphyrinogen IX oxidase
Proto IX: Protoporphyrin IX
RCC: Red Chl catabolite
RCCR: RCC reductase
ROS: Reactive oxygen species
TIC55: Translocon in the inner chloroplast envelope 55
TPB: Tetrapyrrole biosynthesis
UROD: Uroporphyrinogen III decarboxylase
UROS: Uroporphyrinogen III synthetase
UVR8: UV-B resistance 8
